# Nitrous Oxide Gas

**Published:** 1868-06

**Authors:** R. N. Hudson


### ARTICLE IV.

### *Nitrous Oxide Gas.*

By R. N. Hudson, D. D. S.

I will endeavor to give in as concise a manner as possible, information, that I think, must be of the utmost importance, to every member of our profession, and to Surgeons generally, I have been carefully reading every article on the subject, that I could get hold of, hoping for the last six months, that some abler pen than mine, would impart proper information, with regard to this important matter. At the risk of being tedious, permit me to inform you of the efforts I made to arrive at the truth in the premises. Soon after the fall of Richmond, I visited New York City, and made all enquiries relative to all important improvements in the profession. Two Dentists of high standing, told me I had better not touch nitrous oxide gas, it was a humbug, produced spasms in children, and many other dreadful things. I did not touch it for nearly two years, and my commencing the use of it, was almost accidental. Last year I visited Chicago and called on an eminent Dentist; he was administering gas to a lady at the time and by request, I was permitted to witness a very successful operation. He assured me he had used the gas in three thousand cases, with the greatest success. Soon after my return home, I procured an apparatus, and my experience with this anaesthetic agent has been as follows:

Nitrous oxide gas produces a soothing and quieting effect on the most timid and nervous, and after inhaling it a little while, at the request of the operator the mouth will be opened, and the operation submitted to with the greatest willingness—doing away with the necessity of placing a cork between the teeth, and the danger of an overdose, if there is really any danger. The proper moment to commence operating, is a short time after the patient begins to feel numb, and before stertorous breathing commences. I have frequently extracted six to ten teeth, when the patient was in that condition, before the slightest resistance was offered.

I commenced the use of gas last October with a fifty gallon gas apparatus, with Dr. Beans' purifier. My first gas patient was an intelligent youth thirteen or fourteen years of age. I administered the gas to him until he told me he felt a little numb, and cold. I immediately extracted two permanent molars from the lower jaw. He jumped out of the chair delighted saying, "I believe in gas and know perfectly well when you extracted the teeth, but I did not feel the least pain." From that time to the present, I have administered the gas to more than three-hundred persons with similar results in nearly every case, and thus far, I have met with only six persons who could not be affected by it. I think the gas can be kept over lime water for an indefinite period. I kept some of the first gas I made six weeks, and the last gallon of it, was as efficacious and as good as the first. A fifty gallon apparatus is the smallest size that ought to be made. I hope that these suggestions may benefit all of our profession who are desirous of adopting the safest method of alleviating some of the pains of suffering humanity.

